# Atomic Scale Modulation of Self‐Rectifying Resistive Switching by Interfacial Defects

**DOI:** 10.1002/advs.201800096

**Published:** 2018-04-14

**Authors:** Xing Wu, Kaihao Yu, Dongkyu Cha, Michel Bosman, Nagarajan Raghavan, Xixiang Zhang, Kun Li, Qi Liu, Litao Sun, Kinleong Pey

**Affiliations:** ^1^ Division of Microelectronics School of Electrical and Electronic Engineering Nanyang Technological University Singapore 639798 Singapore; ^2^ SEU‐FEI Nano‐Pico Center Key Laboratory of MEMS of Ministry of Education (MOE) Southeast University 2 Sipailou Road Nanjing 210096 China; ^3^ Imaging and Characterization Core Lab 4700 King Abdullah University of Science and Technology Thuwal 23955‐6900 Kingdom of Saudi Arabia; ^4^ Institute of Materials Research and Engineering A*STAR (Agency for Science, Technology and Research) 2 Fusionopolis Way Singapore 138634 Singapore; ^5^ Singapore University of Technology and Design Singapore 487372 Singapore; ^6^ Key Laboratory of Microelectronics Devices & Integration Technology Institute of Microelectronics of Chinese Academy of Sciences Beijing 100029 China

**Keywords:** hafnium dioxide, in situ transmission electron microscopy, interfacial defects, oxygen vacancies, resistive switching

## Abstract

Higher memory density and faster computational performance of resistive switching cells require reliable array‐accessible architecture. However, selecting a designated cell within a crossbar array without interference from sneak path currents through neighboring cells is a general problem. Here, a highly doped n^++^ Si as the bottom electrode with Ni‐electrode/HfO*_x_*/SiO_2_ asymmetric self‐rectifying resistive switching device is fabricated. The interfacial defects in the HfO*_x_*/SiO_2_ junction and n^++^ Si substrate result in the reproducible rectifying behavior. In situ transmission electron microscopy is used to quantitatively study the properties of the morphology, chemistry, and dynamic nucleation–dissolution evolution of the chains of defects at the atomic scale. The spatial and temporal correlation between the concentration of oxygen vacancies and Ni‐rich conductive filament modifies the resistive switching effect. This study has important implications at the array‐level performance of high density resistive switching memories.

## 1 Introduction

Nanocrossbar arrays comprise a set of parallel bottom electrodes and perpendicular top electrodes with a thin layer of resistive switching material in between. Switching materials can be classified into different groups according to their physical mechanisms.^1^, ^2^, ^3^ Materials showing electrochemical metallization or valence change (VCM) effects have been extensively investigated.^4^, ^5^ Each crosspoint in a memory cell stores logic information as a high resistance state (HRS) or a low resistance state (LRS).^6^, ^7^, ^8^ A selection device, such as a transistor or a diode, is usually necessary in addition to the memory element for the scaling limit of the memory device. Therefore, the simplicity of the geometrical structure and the absence of transistors make the concept extremely interesting for low‐power nonvolatile memory circuitry and high integration. Conductive filament (CF) has been recognized as the key structural element that contributed to resistive switching performance.^6^, ^7^, ^9^, ^10^, ^11^ Recently, a number of approaches, such as electrochemical redox reaction,^12^, ^13^ metal nanodots doping,^14^ micrsostructural transitions,^15^, ^16^ and current compliance capping^17^ have been explored to address the controlled formation and rupture mechanism of CFs.

Here in, solving the disadvantage of sneak paths by simply embedding a diode structure inside the memristor cell is proposed. This paper attempts to close the gap in the understanding of the fundamental mechanism through the real‐time observation of resistive switching at an atomic scale. In situ transmission electron microscopy (TEM) technique is a powerful and versatile tool to examine the interfacial properties of the nanodevices.^18^, ^19^, ^20^, ^21^, ^22^ The formation of CF under different compliance current levels controls the growth of the filament. Subsequently, the entire device is switched on and in a cyclic mode to investigate the primary mechanism that leads to a self‐rectifying behavior.

## 2 Results and Discussion

The device used in this study was a unipolar resistive switching device based on an asymmetric metal–insulator–semiconductor (MIS) structure: Ni as the top electrode, HfO_2_ as the insulator, and Si substrate as the bottom electrode. Ni has been used extensively in the mainstream complementary metal oxide semiconductor technology as a source/drain contact material.^23^ The bottom metal electrode was replaced with a highly doped n^++^‐type Si (*N*_D_ ≈10^−19^ cm^3^) to study the role of asymmetric electrode in switching. It comprised of no additional diode and only one resistive switching element (0D1R), which allowed for the construction of highly dense passive crossbar arrays by solving the sneak path problem combined with the drastic reduction of power consumption and area.

**Figure 1:**
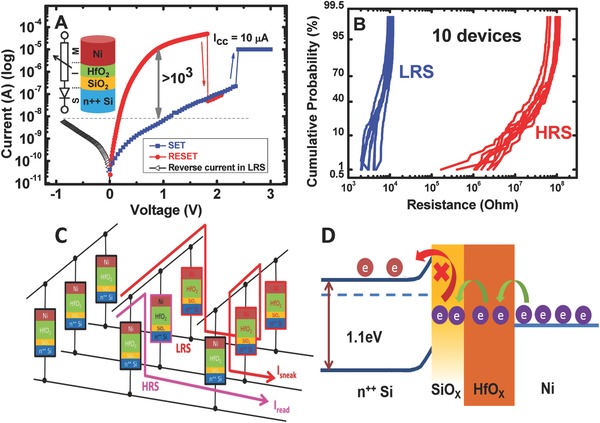
Device electrical performance: A) *I–V* characteristic of a device with MIS structure: Ni/HfO_2_/SiO_2_/Si‐diode with 100 nm Ni, 3.1 nm HfO_2_, and 0.7 nm SiO*_x_*. Note that the reverse current in LRS is very low. The schematic in the inset shows the equivalent circuit after a SET process. B) Device to device resistance distribution in the ON and OFF states for 10 different devices each with 500 DC switching cycles. C) Schematic of an example of a sneak path of a “ON” resistive switching device in a normal circuit. D) Enerrgy band diagram of an “ON” state resistive switching device with negative sweep voltage.

**Figure** **1**A shows that the resistive switching device demonstrates well‐behaved unipolar switching current–voltage (*I–V*) characteristics under DC sweep. The SET voltage was 2.4 V (*V*_SET_) with a compliance current level of 10 µA. Subsequently, the current level reached the 100 µA range with no compliance capping when the voltage was swept again from 0 V with the same polarity. In the RESET sweep, a current drop was observed (three orders of magnitude) at a threshold voltage of 1.8 V (RESET voltage, *V*_RESET_). The HfO_2_‐based resistive switching demonstrated a high ON/OFF resistance ratio of ≈10^3^ with 500 cycles of endurance, as shown in Figure S1 (Supporting Information). Figure 1B shows the device‐to‐device resistance distribution in a Gaussian cumulative density function plot for 10 devices at 500 DC cycles for each device. The LRS had less spread in its resistance value possibly due to the lower variation in the number of defects for the “harder” stages of dielectric breakdown (BD). In contrast, HRS intrinsically had a more extensive spread in its resistance state of ≈2–3 orders of magnitude. This was expected because the CF rupture driven by the local Joule heating effect was difficult to control and occurred within a time range of a few nanoseconds. Therefore, the extent of rupture varied considerably from cycle to cycle. However, die‐to‐die (device‐to‐device) variations become smaller as the distribution tends to overlap very closely with each other. The devices used in this study exhibited good switching parameter uniformity and high‐temperature operating stability. In the crossbar structure‐based memory circuit, all resistive switching devices in a row were connected to each other by the top electrode, and all resistive switching devices in a column were connected to each other by the bottom electrode. The worst case scenario for reading an HRS was assumed, that is, all unselected devices were set to LRS, as illustrated in Figure 1C. Sneak leakage paths^24^ were reduced to a minimum because the reverse current in the LRS of the resistive switching cell in this study under negative voltage sweep was very low (Figure 1A). The low reverse currents can be attributed to the n‐type heavily doped Si bottom electrode that functions as a “diode” in itself, as indicated in Figure 1D. This self‐rectifying effect significantly improved the size of the crossbar array (maximum numbers of rows and columns) because the current flow and voltage drop over the addressed element was strongly dependent on the current flowing through the multiple parallel sneak leakage paths. Furthermore, the overall resistance of the crossbar array depended on the number of “0”s and “1”s stored in the array. Thus, the power consumption decreased when the resistive cells were mainly in the LRS. The integration of a self‐rectifying element addressed the sneak leakage path problem, and this structure is sufficiently scalable. Such devices can also find application in true random number generators, neuromorphic computing and so on.

**Figure 2:**
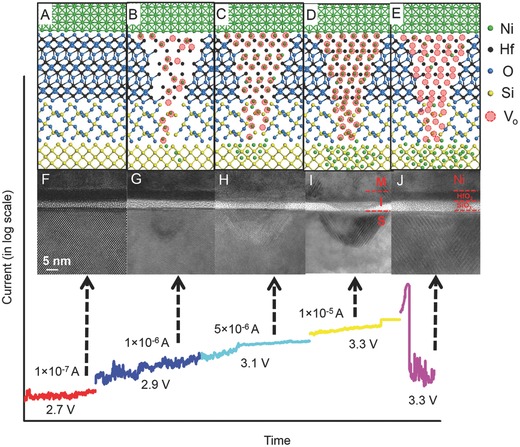
A–J) Evolution of the nanofilament formation and rupture under various compliance currents. Heavy atoms from the top metal electrode migrated into the HfO_2_ dielectric and Si‐substrate, forming a unique geometrical defect which resembles an inverted pyramid if viewed from the Si substrate. Scale bar is 5 nm.

In situ TEM experiments were performed to understand the kinetics of the dynamic nucleation–dissolution behavior of CFs for the *I–V* characteristics shown in Figure 1A. First, the evolution of the formation and rupture of a single nanofilament was studied and a multistage constant voltage stress algorithm developed in ref.^25^ was used. This involved a repetitive sequence of stressing the dielectric from low to high stress voltage conditions at small increments of ≈0.2 V.^25^ This algorithm aims to observe the morphological change of the nanofilament at different stress conditions and identify the critical current levels for the HRS → LRS and LRS → HRS transition. The device was stressed using the proposed test scheme at room temperature^26^ by using an in situ TEM system. In the test, the forming process was initiated with a voltage sweep from 0 to 4.0 V and a compliance setting of 1 µA. The process began with an initial voltage of 2.7 V. It was selected based on a previous study of the gate dielectric BD mechanism.^27^ The in situ stressing voltage was notably higher than the ex situ SET voltage because of the additional parasitic resistance of the in situ TEM tip *R*_tip_ (Figure S2, Supporting Information). The complete current evolution with stress time and the corresponding high‐resolution TEM micrographs are shown in **Figure** **2**A–E. Heavy atoms from the top metal electrode migrated into the HfO_2_ dielectric and Si‐substrate, forming a unique geometrical defect, which resembled an inverted pyramid when viewed from the Si substrate.^28^ A “depleted” region with a lower contrast in the top Ni electrode can be seen on top of the pyramidal (triangle in Figure 2D) defects in the HfO_2_/SiO*_x_* dielectrics, indicating that the Ni atoms from the anode migrated downward. The filament inside the dielectric was bowl‐shaped with a wider top of ≈20 nm near the anode and a shorter bottom of ≈10 nm.^28^ The shape and size of the filaments in the dielectric are consistent with previous observation in both VCM and conductive‐bridge resistive random access memory (CBRAM) reported devices, as reported by pioneering works from authors of refs. 29, 30. Figure 2E shows a RESET stage, where the dark contrast in the dielectric can barely be seen. The device experienced a significant “switch‐off,” which led to a very low current level with an ON/OFF ratio >1000 as shown in Figure 1A,B. The nanofilament was disconnected in the dielectric, and hardly any contrast was detectable inside the HfO_2_ or SiO*_x_* layer. One of the major driving forces that caused the “switch‐off”/rupture of the nanofilament was the Joule heating effect,^31^ which was repeatedly observed in numerous devices, as shown in Figure S3 and Video S3 (Supporting Information). The formation of a nanofilament is associated with the formation of a hillock‐like defects in the Si and SiO_2_ interface. These findings are in agreement with the previous study of dielectric breakdown‐induced epitaxy (DBIE) in an SiO_2_/Si gate stack.^26^, ^27^

**Figure 3:**
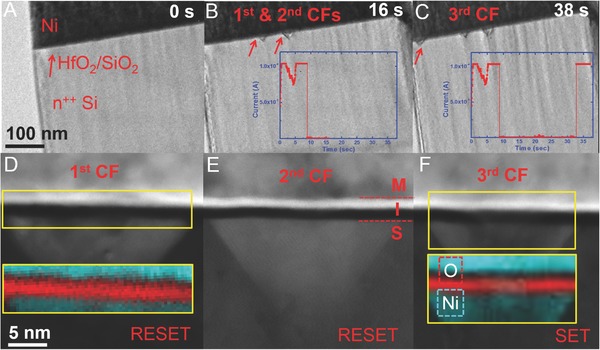
Uncorrelated formation and rupture of multiple nanofilaments under constant voltage stress of 2.7 V. Experiment times: A) 0 s, B) 16 s, the first SET/RESET occurred at ≈1 and ≈2 s and the second SET/RESET happened at ≈7 and ≈9 s. C) After third SET/RESET at ≈38 s in (B). D–F) Corresponding HAADF‐STEM images of the three nanofilaments shown in (B) and (C), respectively. Insets in (D) and (F) are the corresponding EELS mapping results of area of interest of the SET/RESET sites. Cyan represents nickel signals, and red represents oxygen signals, respectively.

Multiple nanofilament formation and rupture events in a single device were also observed in the experiment, as shown in **Figure** **3**. Figure 3A–C shows the dynamic observation of a sequence of morphological changes during the real‐time localized electrical stressing of an MIS resistive switching device in TEM. The first two Ni residuals (1st and 2nd CFs) in the Si substrate (i.e., Figure 3B) indicated the locations of the nanofilaments in two initial cycles of SET and RESET. When the device was switched off for the second time (Figure 3B) at around 9 s for the third SET cycle, a new uncorrelated nanofilament (3rd CF) formed ≈100 nm away from the two previously ruptured filamentary regions (Figure 3C). Figure 3D–F shows the high angle annular dark field (HAADF) scanning transmission electron microscopy (STEM) images (contrast dominated by the atomic number) of the 1st, 2nd, and 3rd nanofilaments, in which the bright contrast in the HfO_2_ and SiO*_x_* layers of the SET filament again indicates that heavy metal atoms from the top metal electrode diffused into the oxide layer. The insets in Figure 3D,F show the Ni and O element electron energy loss spectroscopy (EELS) mapping results. The EELS mapping results confirmed that Ni atoms from the top electrode diffused into the dielectric layers. Ni atoms continued to diffuse along the 〈111〉 direction of the silicon substrate because of the largest inner‐plane distance.^32^ The contrast of each filament can be compared because multiple filaments at the three different locations were observed in a single device. The Ni concentration in the RESET region of the dielectrics was much lower than that in the newly nucleated filament site, which further confirmed the dissolution of a Ni metal filament inside the oxide layer at the nearby SET location.^33^ Video S1 (Supporting Information) shows the dynamic evolution of the process.

**Figure 4:**
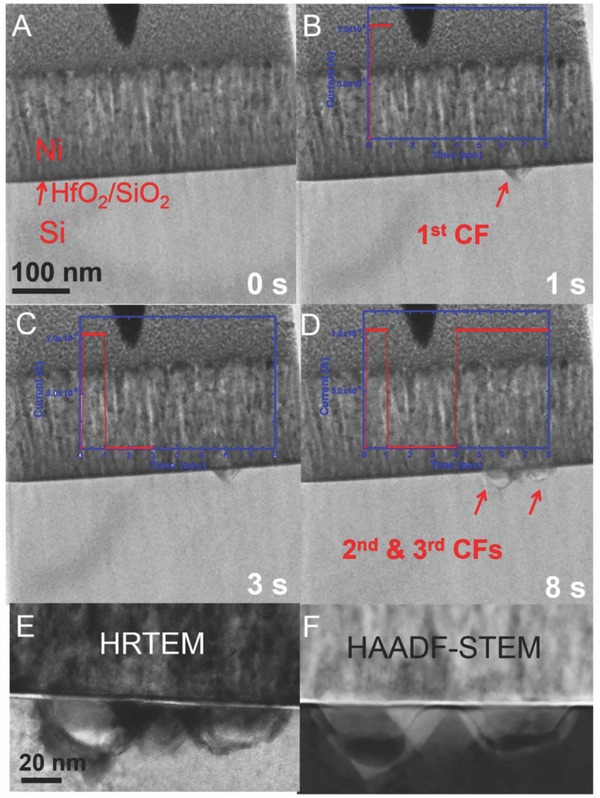
Correlated formation and rupture of multiple nanofilaments. Experiment times: A) 0 s, B) 1 s, C) 3 s, and D) 8 s. E,F) High resolution TEM and HAADF‐STEM micrograph of the formation of two close nanofilaments near the first CF during second SET. Note that there is no heavy atom contrast left in the oxide in the position of the first nanofiament. This verifies that the first nanofilament ruptured quite extensively during the RESET process.

Correlated nanofilament formation and rupture events were also observed. **Figure** **4**A–D shows that the second SET induced two other metal filaments (2nd and 3rd CFs) very near the first one (1st CF) after the first RESET. Even though the first filament experienced substantial rupture of the nanofilament (evident in the significant drop in the switching current), the second nucleation occurred very close to the location of the first one, which can be attributed to the residual metal fragments that remained in the original site after rupture. These Ni residuals served as a weakest link location for subsequent “more localized” switching. Figure 4E,F shows the corresponding high‐resolution TEM and STEM images of the correlated CFs, respectively.

**Figure 5:**
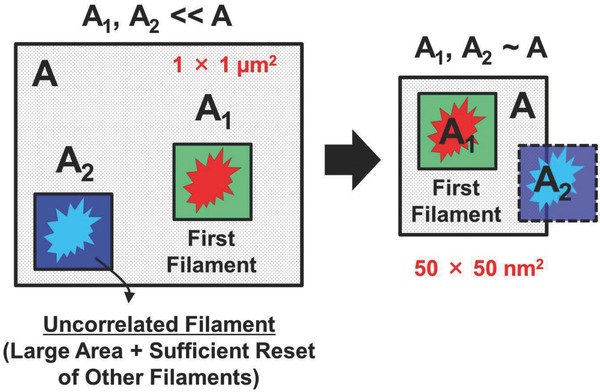
Schematic illustrating the area scaling dependence of multiple filament nucleation probability. The overall area of the resistive random access memory (RRAM) device is denoted as *A*, the first filament area is *A*_1_, and the second filament area is *A*_2_, respectively.

Both single and multiple filaments were typically observed in the in situ TEM experiments. As shown in Figure 4, spatially correlated CFs were regularly observed in some cases of the formation and rupture of multiple filaments. **Figure** **5** presents the schematic of the scaling limitations based on the observed multiple filaments model. The new uncorrelated filaments (*A*_2_) may occur elsewhere in the device if the resistive switching device area (*A*) is much larger than the observed nanofilament size of 20 nm due to the logically higher probability of having multiple weakest link spots in a larger area structure. In this example, the first nanofilament (*A_1_*) is responsible for the SET and RESET which undergoes a very significant formation and rupture of Ni‐filament. Based on Poisson area scaling theory, any new nanofilament could nucleate at other locations for large area devices, as shown in Figure 5. Further experiments, such as a statistical and spatial study of CF distribution, are required to reveal and better understand these underlying mechanisms clearly. Uncorrelated filament formation within a device can lead to unstable state at the LRS and is a challenge for resistive switching application. However, if the reset process is carefully tuned to ensure that the switching occurs at the same place (i.e., energy consumed to switch the same CF is smaller than that for creating a new CF), the variability issue can be better managed. The chances of forming a new filament that is uncorrelated are very low (which is attributed to the area scaling effect in the region of the dielectric that is free of filaments) when the resistive switching area is small. Repeated nucleation at the same nanofilament location or the nucleation of another nanofilament very close to the site of a previous one (“correlated”) could occur. In this scenario, the unit size can be downscaled to the area of a single CF (i.e., about 20 nm, as determined in this study), extensively increasing the cell density and decreasing the energy dissipation for storing a single bit (Video S2, Supporting Information).

## 3 Conclusions

In summary, a high‐performance diode‐free resistive switching cell was fabricated with a high on/off ratio. The real‐time in situ TEM analysis of asymmetric MIS structures allowed the study of the dynamics, evolution, and underlying physics governing the switching mechanism, which was previously speculated at best from electrical measurements without much direct evidence. Multiple conductive path formation and rupture events were temporally and spatially uncorrelated. The use of an asymmetric MIS structure clearly assisted in identifying the presence and source of the metal‐rich conductive filament, which originated from the anode electrode while preventing sneak path issues. This fundamental atomic study on the chemistry, morphology, and time‐dependent correlated/uncorrelated switching behavior of filaments provides a strong support to the feasibility of further scaling of future resistive switching technologies, paving the way for very high‐density data storage and future neuromorphic computing.

## Experimental Section

*Electrical Characterization*: The electrical performance of the device was determined ex situ using a probe station with the standard Keithley SCS‐4200 semiconductor characterization system at room temperature.

*FIB‐SEM Sample Preparation*: The device was prepared using a focused ion beam using a gallium beam in a dual‐beam Helios 600i system from FEI. A Low‐kV (2 kV) cleaning process was used to remove the surface amorphous layer. Finally, the thickness of the TEM lamella was reduced to ≈40 nm by FIB cutting. Each resistive switching device was isolated by ion milling.

*In Situ TEM*: The TEM instrument was a Cs‐corrected TEM (FEI Titan 80–300) with an operation acceleration voltage of 200 kV. The electron beam was carefully spread out to avoid any e‐beam‐induced structural damage while maintaining atomic resolution. EELS signals were collected under an 80 kV e‐beam rather than a 200 kV e‐beam to ensure that no sample damage occurred because of long electron irradiation time. Elemental maps were estimated by determining the absorption edge area after a power law background subtraction. Edge area densities were estimated using the formula *NI*_Total_ = *I*_k_/σ_k_, where *N* is the atomic concentration, and *I*_k_ and σ_k_ are the intensity and ionization cross‐sections of the absorption edge, respectively. A chemical sensitivity of 0.1% was achieved. STEM–EELS chemical maps were recorded with an energy resolution of 0.7 eV and a spatial resolution of 0.3 nm.

## Conflict of Interest

The authors declare no conflict of interest.

## Supporting information

Supplementary

Supplementary

Supplementary

Supplementary
