# Supplementary material for: Atomic Scale Modulation of Self‐Rectifying Resistive Switching by Interfacial Defects
Source: Adv Sci (Weinh). 2018 Apr 14;5(6):1800096. doi: 10.1002/advs.201800096 (PMC6010905; doi:10.1002/advs.201800096)
Supplement: Supplementary file 1 — Supplementary [file ADVS-5-1800096-s002.pdf]

Copyright WILEY-VCH Verlag GmbH & Co. KGaA, 69469 Weinheim, Germany, 2018.

ADVANCED SCIENCE
Open Access

Supporting Information

for *Adv. Sci.,* DOI: 10.1002/advs.201800096

Atomic Scale Modulation of Self-Rectifying Resistive Switching by Interfacial Defects

Xing Wu, Kaihao Yu, Dongkyu Cha, Michel Bosman, Nagarajan Raghavan, Xixiang Zhang, Kun Li, Qi Liu, Litao Sun,* and Kinleong Pey**

Copyright WILEY-VCH Verlag GmbH & Co. KGaA, 69469 Weinheim, Germany, 2016.

Supporting Information

Atomic scale modulation of self-rectifying resistive switching by interfacial defects

*Xing Wu**, *Kaihao Yu, Dongkyu Cha, Michel Bosman, Nagarajan Raghavan, Xixiang Zhang, Kun Li, Qi Liu, Litao Sun**, *Kinleong Pey**

Dr. X. Wu, Dr. N. Raghavan, Prof. K. L. Pey

Division of Microelectronics, School of Electrical and Electronic Engineering, Nanyang Technological University, Singapore 639798, Singapore

Email: xwu@ee.ecnu.edu.cn

Dr. X. Wu, K. H. Yu, Prof. L. T. Sun

SEU-FEI Nano-Pico Center, Key Laboratory of MEMS of Ministry of Education (MOE), Southeast University, 2 Sipailou Road, Nanjing 210096, China

Email: slt@seu.edu.cn

Dr. D. Cha, Prof. K. Li, Prof. X. X. Zhang

Imaging and Characterization Core Lab, 4700 King Abdullah University of Science and Technology, Thuwal 23955-6900, Kingdom of Saudi Arabia

Dr. M. Bosman

Institute of Materials Research and Engineering, Agency for Science, Technology and Research, 3 Research Link, Singapore 117602, Singapore.

Prof. Q. Liu

Key Laboratory of Microelectronics Devices & Integration Technology, Institute of Microelectronics of Chinese Academy of Sciences, Beijing 100029, China

Dr. N. Raghavan, Prof. K. L. Pey

Singapore University of Technology and Design, Singapore 487372, Singapore.

Email: peykinleong@sutd.edu.sg

These authors contributed equally: Xing Wu, Kaihao Yu, and Dongkyu Cha

*These corresponding authors contributed equally: xwu@ee.ecnu.edu.cn; slt@seu.edu.cn; peykinleong@sutd.edu.sg

Supplementary Movies

Movie S1: This video shows a case of uncorrelated formation and rupture of multiple nanofilaments under CVS of 2.7 V.

Movie S2: This video shows a case of correlated formation and rupture of multiple nanofilaments.

Movie S3: This video shows the interfacial defects evolution of a single nanofilament.

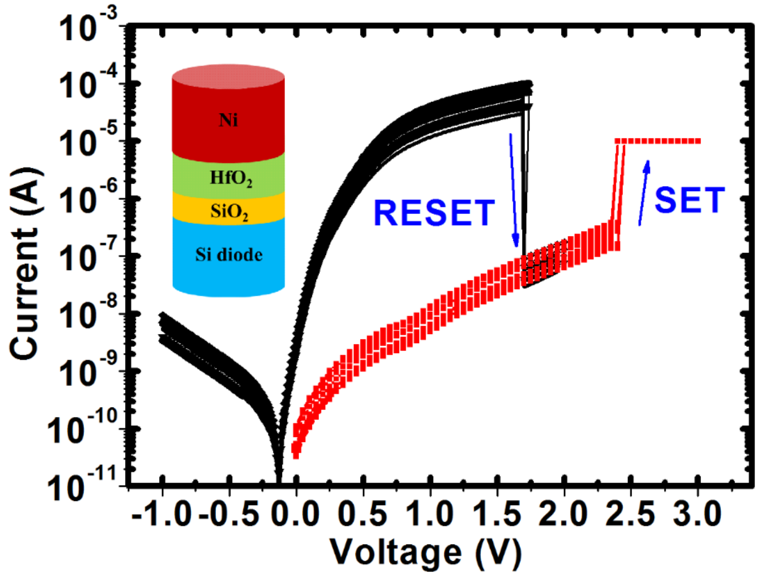

Figure S1: Switching characteristics of the MIS RRAM device for 500 SET – RESET cycles using DC sweep. The inset shows the schematic of the device.

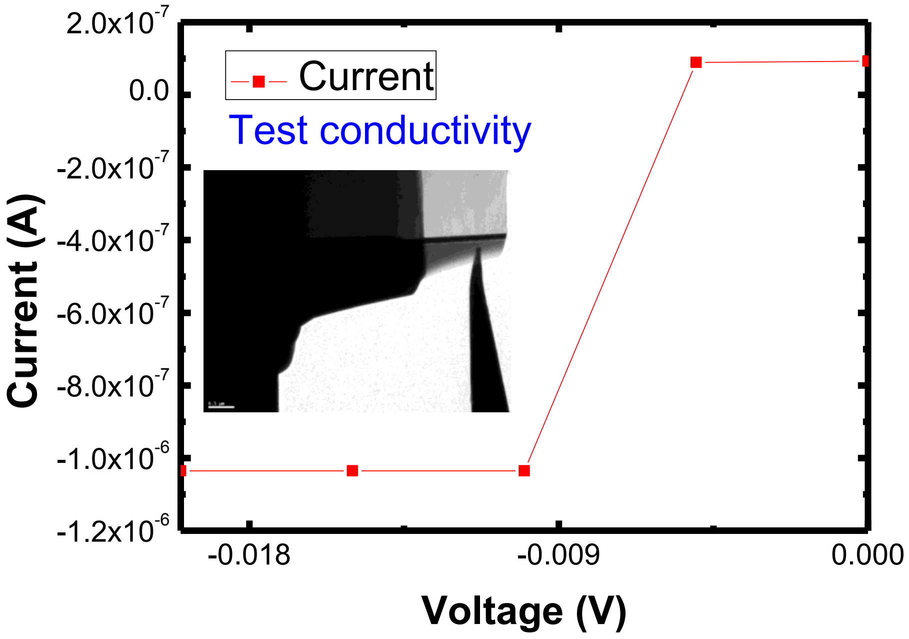

Figure S2: In situ W tip conductivity test. Inset is the TEM image of the system. The

resistance of W tip, R_{tip} is ~5-8 kΩ.

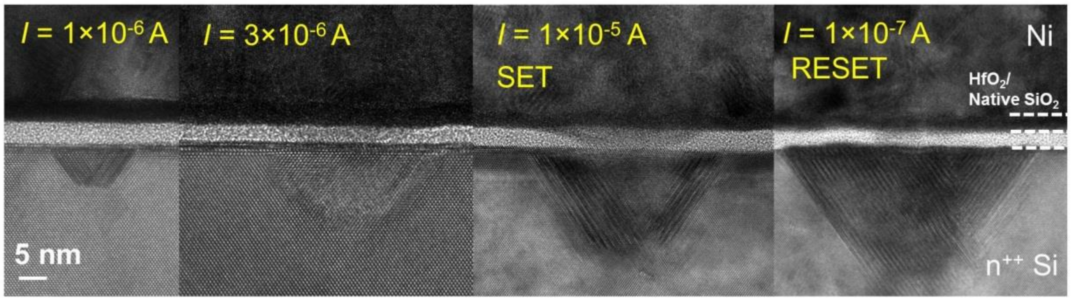

Figure S3: Evolution of the nanofilament formation and rupture under various compliance currents. Scale bar is 5 nm.
